# 3,4,5-Trimeth­oxy-4′-methyl­biphen­yl

**DOI:** 10.1107/S1600536813010969

**Published:** 2013-04-30

**Authors:** Manu Lahtinen, Kalle Nättinen, Sami Nummelin

**Affiliations:** a University of Jyväskylä, Department of Chemistry, PO Box 35, FI-40014 JY, Finland; b VTT Technical Research Centre of Finland, Tampere, FIN-33101, Finland; c Molecular Materials, Department of Applied Physics, School of Science, Aalto University, PO Box 15100, FI-00076 Aalto, Finland

## Abstract

In the title compound, C_16_H_18_O_3_, the dihedral angle between the benzene rings is 33.4 (2)°. In the crystal, mol­ecules are packed in a zigzag arrangement along the *b*-axis and are inter­connected *via* weak C—H⋯O hydrogen bonds, and C—H⋯π inter­actions involving the meth­oxy groups and the benzene rings of neighbouring molecules.

## Related literature

For related single-crystal structures based on AB_2_– and AB_3_-branched bi­phenyls, see: Lahtinen *et al.* (2013*a* ▶,*b* ▶,*c* ▶); Lahtinen & Nummelin (2013 ▶). For synthesis of the title compound, see: Percec *et al.* (2006 ▶, 2007 ▶). For crystal structures of dendrimers, see: Mekelburger *et al.* (1993 ▶); Nättinen & Rissanen (2003 ▶); Ropponen *et al.* (2004*a* ▶). For related Percec-type self-assembling supra­molecular dendrimers, see: Percec *et al.* (2006 ▶, 2007 ▶, 2008 ▶); Roche & Percec (2013 ▶). For dendrimersomes, see: Percec *et al.* (2010 ▶). For aliphatic and aromatic polyester building blocks for dendrimersomes, see: Ropponen *et al.* (2004**b* ▶,c* ▶); Nummelin *et al.* (2000 ▶).
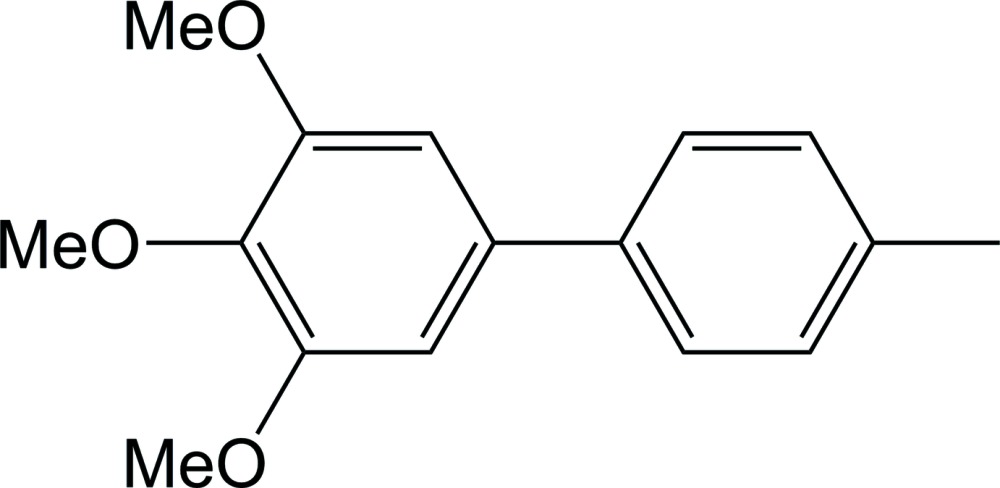


## Experimental

### 

#### Crystal data

C_16_H_18_O_3_
*M* *_r_* = 258.30
Orthorhombic, 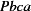

*a* = 8.4669 (2) Å
*b* = 15.0636 (3) Å
*c* = 21.4516 (4) Å
*V* = 2735.98 (10) Å^3^
*Z* = 8
Mo *K*α radiation
μ = 0.09 mm^−1^
*T* = 173 K
0.3 × 0.25 × 0.2 mm

#### Data collection

Bruker–Nonius KappaCCD diffractometer equipped with an APEXII detector
Absorption correction: multi-scan (*SADABS*; Sheldrick, 1996 ▶) *T*_min_ = 0.975, *T*_max_ = 0.983
17856 measured reflections
2589 independent reflections
1984 reflections with *I* > 2σ(*I*)
*R*_int_ = 0.052

#### Refinement

*R*[*F* ^2^ > 2σ(*F* ^2^)] = 0.043
*wR*(*F* ^2^) = 0.117
*S* = 1.03
2589 reflections
173 parameters
H-atom parameters constrained
Δρ_max_ = 0.28 e Å^−3^
Δρ_min_ = −0.20 e Å^−3^

### 

Data collection: *COLLECT* (Nonius, 1998 ▶); cell refinement: *SCALEPACK* (Otwinowski & Minor, 1997 ▶); data reduction: *DENZO* (Otwinowski & Minor, 1997 ▶) and *SCALEPACK*; program(s) used to solve structure: *SHELXS97* (Sheldrick, 2008 ▶); program(s) used to refine structure: *SHELXL97* (Sheldrick, 2008 ▶); molecular graphics: *OLEX2* (Dolomanov *et al.*, 2009 ▶) and *Mercury* (Macrae *et al.*, 2006 ▶); software used to prepare material for publication: *OLEX2*.

## Supplementary Material

Crystal structure: contains datablock(s) I, global. DOI: 10.1107/S1600536813010969/go2088sup1.cif

Structure factors: contains datablock(s) I. DOI: 10.1107/S1600536813010969/go2088Isup2.hkl

Supplementary material file. DOI: 10.1107/S1600536813010969/go2088Isup3.cml

Additional supplementary materials:  crystallographic information; 3D view; checkCIF report

## Figures and Tables

**Table 1:** Hydrogen-bond geometry (Å, °) *Cg*1 and *Cg*2 are the centroids of the C2–C7 and C8–C10/C13/C16/C19 aromatic rings, respectively.

*D*—H⋯*A*	*D*—H	H⋯*A*	*D*⋯*A*	*D*—H⋯*A*
C4—H4⋯O11^i^	0.95	2.57	3.382 (2)	144
C12—H12*B*⋯O14^i^	0.98	2.56	3.465 (2)	154
C18—H18*C*⋯O17^ii^	0.98	2.63	3.488 (2)	146
C15—H15*A*⋯*Cg*1^iii^	0.98	2.84	3.692 (2)	139
C12—H12*A*⋯*Cg*2^iv^	0.98	3.19	4.061 (2)	132
C18—H18*B*⋯*Cg*1^v^	0.98	3.01	3.976 (2)	149

Symmetry codes: (i) 
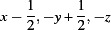
; (ii) 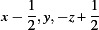; (iii) 
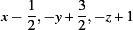
; (iv) 
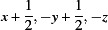
; (v) 
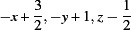
.

**Fig. 1.**
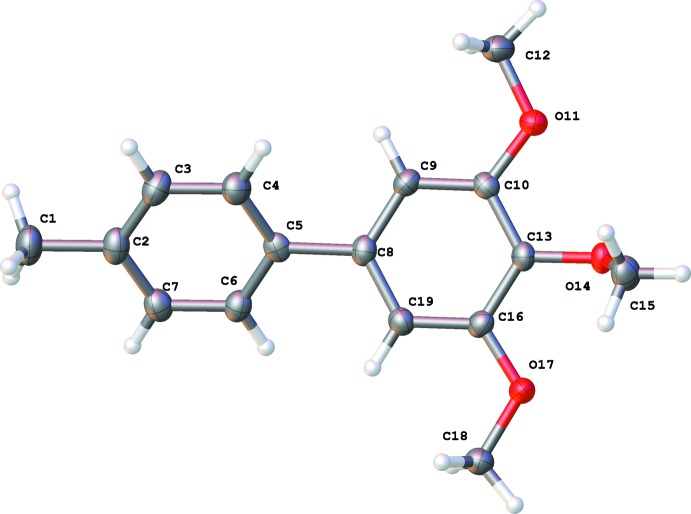
The molecular structure and atomic numbering of the title compound showing 50% probability displacement ellipsoids.

**Fig. 2.**
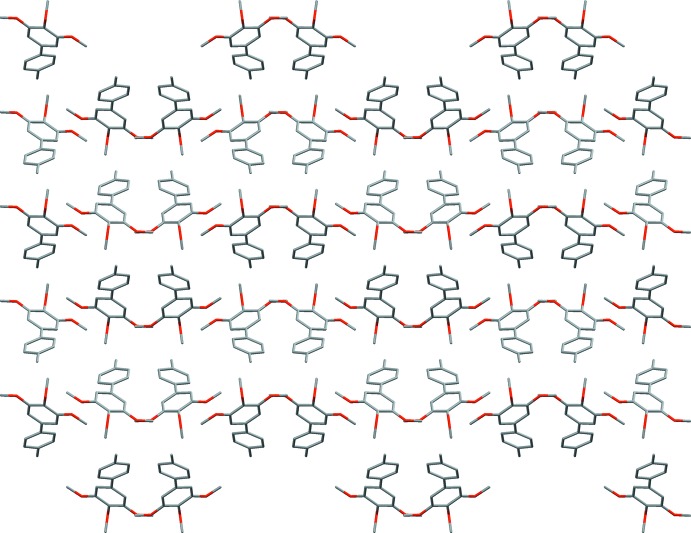
Antiparallel rows of molecules viewed along a-axis.

**Fig. 3.**
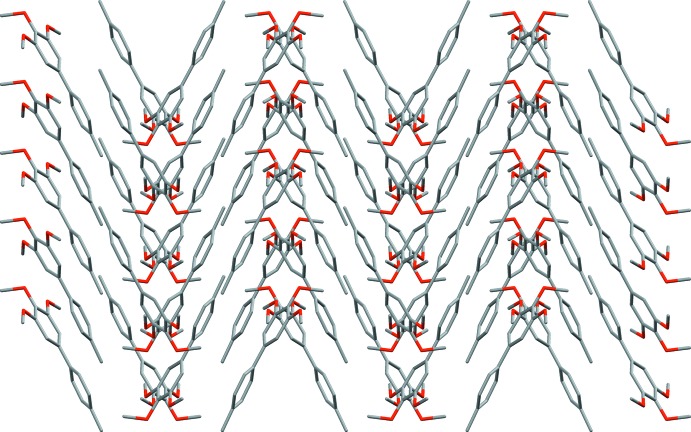
The zigzag arrangement of the molecules viewed along c-axis.

**Fig. 4.**
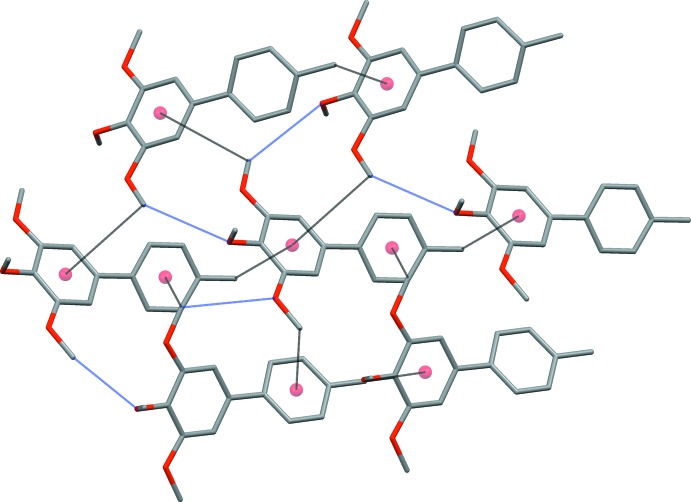
CH···O and CH···π interactions shown by blue and black contact lines, respectively.

## supplementary crystallographic information

### Comment

3,4,5-Trimethoxy-4'-methyl biphenyl was synthesized in a gram quantities by employing a metal catalyzed coupling reaction between an aryl bromide and *p*-tolylboronic acid (Percec *et al.* 2006, 2007). The title compound (I) was used as a building block for the construction of amphiphilic AB_2_– and AB_3_-branched biphenyl dendrons (Percec *et al.* 2006) and hybrid (phenyl–biphenyl) dendrons (Percec *et al.* 2007). With few exceptions (*e.g.* Mekelburger *et al.* 1993; Nättinen & Rissanen 2003; Ropponen *et al.* 2004*a*) most dendrimers are liquid or amorphous. However, Percec-type dendrons and dendrimers have the ability to self-assemble in the solid state and in selected solvents into supramolecular architectures, such as hollow or non-hollow columns or spheres, which, in turn, self-organize into periodic lattices or quasi-periodic arrays in the solid state (Percec *et al.* 2006, 2007, 2008). In addition, biphenyls (Percec *et al.* 2006, 2007) are key building blocks on expanding the scope of libraries of amphiphilic Janus-dendrimers (Ropponen *et al.* 2004*b*; Percec *et al.* 2010) based on hydrophobic Percec-type building blocks and hydrophilic aliphatic and aromatic polyester building blocks. (Ropponen *et al.* 2004**b*,c*; Nummelin *et al.* 2000). Amphiphilic Janus-dendrimers self-assemble into uniform liposome-like structures denoted as *dendrimersomes* (Percec *et al.* 2010) and other complex adaptable systems (Roche & Percec 2013) in water and selected biological buffers. Herein, we report the title compound 3,4,5-trimethoxy-4'-methyl biphenyl (I) as a contribution to a structural study of biphenyl derivatives (Lahtinen *et al.* 2013*a*,*b*,*c*; Lahtinen & Nummelin 2013).

Compound (I) has a dihedral angle between the aromatic rings of 33.4 (2)°, and is analogous to various biphenyl structures (Lahtinen *et al.* 2103*a*,*b*). The methoxy groups in 3- and 5-positions (Fig. 1) are co-planar with the [C(8)>C(19)] ring with the dihedral angles of 0.2 (2)° and 0.7 (2)°, respectively, whereas the methoxy group in the 4-position is tilted out from the plane with angle 113.32 (13)°. The molecules are packed in a zigzag formation along *b* -axis. This formation origates from antiparallel rows of molecules running through *c* -axis (Figures 2 and 3). Three weak CH···O hydrogen bonds occur with donor-acceptor d(D···A) bond distances of 3.382 (2), 3.465 (2), and 3.488 (2) Å, respectively (Fig. 4). Moreover, a network of weak CH···π interactions is observable between methoxy groups and nearby phenyl groups having ring-centroid to methyl(C) distances of 3.692 (2) - 4.061 (2)Å.

### Experimental

A flame-dried Schlenk-tube was loaded with *p*-tolylboronic acid (3.3 g, 24.3 mmol), KF (2.8 g, 48.6 mmol), 3,4,5-trimethoxy bromobenzene (4.0 g, 16.2 mmol), Pd(OAc)_2_ (36 mg, 0.16 mmol, 1.0 mol%) and 2-(di-*tert*-butylphosphino)biphenyl (97 mg, 0.33 mmol, 2.0 mol%). The tube was sealed with a teflon screwcap and evacuated/backfilled with argon (5x). Then dry, degassed THF (30 ml) was added *via* syringe and the reaction mixture was stirred at RT until the aryl bromide had been completely consumed as judged by TLC analysis. The mixture was diluted with ether, filtered, and washed with 1*M* NaOH. The aqueous layer was extracted with ether, the combined organic layer was washed with brine and dried with MgSO_4_. After evaporation the pale yellow solid was chromatographed on silica gel using dichloromethane as eluent. Recrystallization from ethanol gave 3.9 g (93%) of the title compound (I) as a white crystalline solid. Crystals suitable for a single-crystal structure determination were obtained from a slow evaporation of the solvent.

### Refinement

Hydrogen atoms were calculated to their positions as riding atoms (C host) using isotropic displacement parameters that were fixed to be 1.2 or 1.5 times larger than those of the attached non-hydrogen atom.

### Crystal data

**Table:**

C_16_H_18_O_3_	*D*_x_ = 1.254 Mg m^−^^3^
*M**_r_* = 258.30	Mo *K*α radiation, λ = 0.71069 Å
Orthorhombic, *P**b**c**a*	Cell parameters from 9816 reflections
*a* = 8.4669 (2) Å	θ = 2.9–25.7°
*b* = 15.0636 (3) Å	µ = 0.09 mm^−^^1^
*c* = 21.4516 (4) Å	*T* = 173 K
*V* = 2735.98 (10) Å^3^	Plate, colourless
*Z* = 8	0.3 × 0.25 × 0.2 mm
*F*(000) = 1104	

### Data collection

**Table:**

Bruker–Nonius KappaCCD diffractometer equipped with an APEXII detector	2589 independent reflections
Radiation source: sealed tube	1984 reflections with *I* > 2σ(*I*)
Graphite monochromator	*R*_int_ = 0.052
Detector resolution: 8 pixels mm^-1^	θ_max_ = 25.7°, θ_min_ = 2.9°
ω and φ scans	*h* = −10→10
Absorption correction: multi-scan (*SADABS*; Sheldrick, 1996)	*k* = −16→18
*T*_min_ = 0.975, *T*_max_ = 0.983	*l* = −25→26
17856 measured reflections	

### Refinement

**Table:**

Refinement on *F*^2^	Primary atom site location: structure-invariant direct methods
Least-squares matrix: full	Secondary atom site location: difference Fourier map
*R*[*F*^2^ > 2σ(*F*^2^)] = 0.043	Hydrogen site location: inferred from neighbouring sites
*wR*(*F*^2^) = 0.117	H-atom parameters constrained
*S* = 1.03	*w* = 1/[σ^2^(*F*_o_^2^) + (0.0571*P*)^2^ + 1.2608*P*] where *P* = (*F*_o_^2^ + 2*F*_c_^2^)/3
2589 reflections	(Δ/σ)_max_ < 0.001
173 parameters	Δρ_max_ = 0.28 e Å^−^^3^
0 restraints	Δρ_min_ = −0.20 e Å^−^^3^
0 constraints	

### Special details

**Table:**

Geometry. All e.s.d.'s (except the e.s.d. in the dihedral angle between two l.s. planes) are estimated using the full covariance matrix. The cell e.s.d.'s are taken into account individually in the estimation of e.s.d.'s in distances, angles and torsion angles; correlations between e.s.d.'s in cell parameters are only used when they are defined by crystal symmetry. An approximate (isotropic) treatment of cell e.s.d.'s is used for estimating e.s.d.'s involving l.s. planes.
Refinement. Refinement of *F*^2^ against ALL reflections. The weighted *R*-factor *wR* and goodness of fit *S* are based on *F*^2^, conventional *R*-factors *R* are based on *F*, with *F* set to zero for negative *F*^2^. The threshold expression of *F*^2^ > σ(*F*^2^) is used only for calculating *R*-factors(gt) *etc*. and is not relevant to the choice of reflections for refinement. *R*-factors based on *F*^2^ are statistically about twice as large as those based on *F*, and *R*- factors based on ALL data will be even larger.

### Fractional atomic coordinates and isotropic or equivalent isotropic displacement parameters (Å^2^)

**Table:**

	*x*	*y*	*z*	*U*_iso_*/*U*_eq_	
C1	−0.5594 (2)	−0.00288 (14)	0.14685 (10)	0.0423 (5)	
H1A	−0.6400	0.0432	0.1521	0.063*	
H1B	−0.5619	−0.0432	0.1827	0.063*	
H1C	−0.5807	−0.0364	0.1086	0.063*	
C2	−0.3984 (2)	0.03996 (12)	0.14242 (9)	0.0324 (4)	
C3	−0.3444 (2)	0.07517 (12)	0.08647 (9)	0.0333 (4)	
H3	−0.4095	0.0715	0.0505	0.040*	
C4	−0.1974 (2)	0.11563 (11)	0.08190 (8)	0.0305 (4)	
H4	−0.1630	0.1387	0.0430	0.037*	
C5	−0.10199 (19)	0.12239 (10)	0.13322 (7)	0.0230 (4)	
C6	−0.1534 (2)	0.08818 (12)	0.18971 (8)	0.0284 (4)	
H6	−0.0881	0.0929	0.2256	0.034*	
C7	−0.2995 (2)	0.04713 (11)	0.19405 (8)	0.0311 (4)	
H7	−0.3327	0.0235	0.2329	0.037*	
C8	0.0553 (2)	0.17020 (11)	0.12955 (8)	0.0245 (4)	
C9	0.1441 (2)	0.16780 (11)	0.07478 (7)	0.0248 (4)	
H9	0.1084	0.1337	0.0403	0.030*	
C10	0.28466 (19)	0.21507 (11)	0.07052 (7)	0.0229 (4)	
C12	0.3296 (2)	0.16804 (13)	−0.03432 (8)	0.0356 (5)	
H12A	0.4071	0.1748	−0.0679	0.053*	
H12B	0.2268	0.1904	−0.0483	0.053*	
H12C	0.3201	0.1052	−0.0232	0.053*	
C13	0.33806 (19)	0.26545 (11)	0.12094 (7)	0.0233 (4)	
C15	0.4574 (2)	0.40464 (12)	0.10715 (9)	0.0347 (4)	
H15A	0.5609	0.4335	0.1040	0.052*	
H15B	0.3993	0.4294	0.1426	0.052*	
H15C	0.3977	0.4150	0.0687	0.052*	
C16	0.2514 (2)	0.26602 (11)	0.17617 (7)	0.0244 (4)	
C18	0.2258 (2)	0.31828 (12)	0.28086 (7)	0.0294 (4)	
H18A	0.2816	0.3556	0.3112	0.044*	
H18B	0.2148	0.2581	0.2976	0.044*	
H18C	0.1209	0.3433	0.2728	0.044*	
C19	0.1099 (2)	0.21951 (11)	0.18045 (8)	0.0251 (4)	
H19	0.0502	0.2212	0.2179	0.030*	
O11	0.38021 (14)	0.21736 (8)	0.01893 (5)	0.0286 (3)	
O14	0.47847 (13)	0.31138 (8)	0.11607 (5)	0.0270 (3)	
O17	0.31410 (14)	0.31530 (8)	0.22369 (5)	0.0309 (3)	

### Atomic displacement parameters (Å^2^)

**Table:**

	*U*^11^	*U*^22^	*U*^33^	*U*^12^	*U*^13^	*U*^23^
C1	0.0279 (10)	0.0437 (12)	0.0554 (13)	−0.0092 (9)	0.0020 (9)	−0.0026 (10)
C2	0.0239 (9)	0.0260 (9)	0.0473 (11)	−0.0003 (8)	0.0001 (8)	−0.0004 (8)
C3	0.0298 (10)	0.0290 (10)	0.0413 (10)	−0.0032 (8)	−0.0080 (8)	0.0006 (8)
C4	0.0309 (10)	0.0260 (9)	0.0347 (9)	−0.0022 (8)	0.0003 (8)	0.0020 (8)
C5	0.0222 (9)	0.0167 (8)	0.0302 (8)	0.0055 (7)	0.0031 (7)	0.0009 (6)
C6	0.0242 (9)	0.0265 (9)	0.0346 (9)	−0.0002 (7)	−0.0020 (7)	−0.0013 (7)
C7	0.0294 (10)	0.0273 (10)	0.0366 (10)	−0.0011 (8)	0.0050 (8)	0.0009 (8)
C8	0.0201 (9)	0.0225 (8)	0.0307 (9)	0.0008 (7)	−0.0023 (7)	0.0020 (7)
C9	0.0251 (9)	0.0242 (9)	0.0252 (8)	−0.0010 (7)	−0.0038 (7)	−0.0020 (7)
C10	0.0218 (9)	0.0241 (9)	0.0229 (8)	0.0025 (7)	0.0009 (6)	0.0027 (6)
C12	0.0410 (11)	0.0395 (11)	0.0262 (9)	−0.0062 (9)	0.0034 (8)	−0.0062 (8)
C13	0.0173 (8)	0.0243 (9)	0.0283 (9)	0.0003 (7)	−0.0006 (6)	0.0013 (7)
C15	0.0336 (11)	0.0278 (10)	0.0426 (11)	−0.0050 (8)	0.0047 (9)	−0.0012 (8)
C16	0.0213 (8)	0.0259 (9)	0.0259 (8)	0.0011 (7)	−0.0029 (7)	−0.0034 (7)
C18	0.0287 (10)	0.0335 (10)	0.0259 (9)	−0.0017 (8)	0.0027 (7)	−0.0037 (7)
C19	0.0219 (9)	0.0272 (9)	0.0261 (9)	0.0001 (7)	0.0018 (7)	0.0002 (7)
O11	0.0276 (7)	0.0353 (7)	0.0229 (6)	−0.0037 (5)	0.0021 (5)	−0.0031 (5)
O14	0.0191 (6)	0.0283 (7)	0.0335 (7)	−0.0037 (5)	0.0015 (5)	−0.0025 (5)
O17	0.0247 (6)	0.0421 (8)	0.0259 (6)	−0.0074 (6)	0.0028 (5)	−0.0094 (5)

### Geometric parameters (Å, º)

**Table:**

C1—H1A	0.9800	C10—C13	1.396 (2)
C1—H1B	0.9800	C10—O11	1.3713 (19)
C1—H1C	0.9800	C12—H12A	0.9800
C1—C2	1.511 (3)	C12—H12B	0.9800
C2—C3	1.389 (3)	C12—H12C	0.9800
C2—C7	1.393 (3)	C12—O11	1.429 (2)
C3—H3	0.9500	C13—C16	1.394 (2)
C3—C4	1.389 (3)	C13—O14	1.3795 (19)
C4—H4	0.9500	C15—H15A	0.9800
C4—C5	1.370 (2)	C15—H15B	0.9800
C5—C6	1.387 (2)	C15—H15C	0.9800
C5—C8	1.516 (2)	C15—O14	1.429 (2)
C6—H6	0.9500	C16—C19	1.391 (2)
C6—C7	1.386 (3)	C16—O17	1.3683 (19)
C7—H7	0.9500	C18—H18A	0.9800
C8—C9	1.396 (2)	C18—H18B	0.9800
C8—C19	1.399 (2)	C18—H18C	0.9800
C9—H9	0.9500	C18—O17	1.4370 (19)
C9—C10	1.389 (2)	C19—H19	0.9500
			
H1A—C1—H1B	109.5	O11—C10—C13	114.86 (14)
H1A—C1—H1C	109.5	H12A—C12—H12B	109.5
H1B—C1—H1C	109.5	H12A—C12—H12C	109.5
C2—C1—H1A	109.5	H12B—C12—H12C	109.5
C2—C1—H1B	109.5	O11—C12—H12A	109.5
C2—C1—H1C	109.5	O11—C12—H12B	109.5
C3—C2—C1	120.94 (17)	O11—C12—H12C	109.5
C3—C2—C7	117.35 (16)	C16—C13—C10	119.42 (15)
C7—C2—C1	121.70 (17)	O14—C13—C10	119.53 (14)
C2—C3—H3	119.2	O14—C13—C16	121.01 (14)
C4—C3—C2	121.55 (17)	H15A—C15—H15B	109.5
C4—C3—H3	119.2	H15A—C15—H15C	109.5
C3—C4—H4	119.9	H15B—C15—H15C	109.5
C5—C4—C3	120.30 (17)	O14—C15—H15A	109.5
C5—C4—H4	119.9	O14—C15—H15B	109.5
C4—C5—C6	119.32 (16)	O14—C15—H15C	109.5
C4—C5—C8	120.80 (15)	C19—C16—C13	120.44 (15)
C6—C5—C8	119.83 (15)	O17—C16—C13	115.61 (15)
C5—C6—H6	119.9	O17—C16—C19	123.95 (15)
C7—C6—C5	120.29 (16)	H18A—C18—H18B	109.5
C7—C6—H6	119.9	H18A—C18—H18C	109.5
C2—C7—H7	119.4	H18B—C18—H18C	109.5
C6—C7—C2	121.19 (17)	O17—C18—H18A	109.5
C6—C7—H7	119.4	O17—C18—H18B	109.5
C9—C8—C5	120.31 (14)	O17—C18—H18C	109.5
C9—C8—C19	119.55 (15)	C8—C19—H19	120.0
C19—C8—C5	120.11 (15)	C16—C19—C8	120.02 (15)
C8—C9—H9	119.9	C16—C19—H19	120.0
C10—C9—C8	120.22 (15)	C10—O11—C12	117.08 (13)
C10—C9—H9	119.9	C13—O14—C15	113.32 (13)
C9—C10—C13	120.32 (15)	C16—O17—C18	116.80 (13)
O11—C10—C9	124.82 (14)		
			
C1—C2—C3—C4	179.25 (17)	C9—C8—C19—C16	0.3 (2)
C1—C2—C7—C6	−178.67 (17)	C9—C10—C13—C16	1.7 (2)
C2—C3—C4—C5	−0.5 (3)	C9—C10—C13—O14	179.48 (14)
C3—C2—C7—C6	0.3 (3)	C9—C10—O11—C12	0.7 (2)
C3—C4—C5—C6	0.2 (3)	C10—C13—C16—C19	−2.3 (2)
C3—C4—C5—C8	−177.12 (16)	C10—C13—C16—O17	178.43 (15)
C4—C5—C6—C7	0.4 (3)	C10—C13—O14—C15	104.14 (17)
C4—C5—C8—C9	−33.4 (2)	C13—C10—O11—C12	−179.01 (15)
C4—C5—C8—C19	144.48 (17)	C13—C16—C19—C8	1.4 (3)
C5—C6—C7—C2	−0.6 (3)	C13—C16—O17—C18	178.99 (15)
C5—C8—C9—C10	176.97 (15)	C16—C13—O14—C15	−78.13 (19)
C5—C8—C19—C16	−177.60 (15)	C19—C8—C9—C10	−0.9 (2)
C6—C5—C8—C9	149.32 (16)	C19—C16—O17—C18	−0.2 (2)
C6—C5—C8—C19	−32.8 (2)	O11—C10—C13—C16	−178.55 (14)
C7—C2—C3—C4	0.3 (3)	O11—C10—C13—O14	−0.8 (2)
C8—C5—C6—C7	177.71 (15)	O14—C13—C16—C19	179.92 (15)
C8—C9—C10—C13	−0.1 (2)	O14—C13—C16—O17	0.7 (2)
C8—C9—C10—O11	−179.81 (15)	O17—C16—C19—C8	−179.47 (15)

### Hydrogen-bond geometry (Å, º)

Cg1 and Cg2 are the centroids of the C2–C7 and C8–C10/C13/C16/C19 aromatic rings, respectively.

**Table:**

*D*—H···*A*	*D*—H	H···*A*	*D*···*A*	*D*—H···*A*
C4—H4···O11^i^	0.95	2.57	3.382 (2)	144
C12—H12*B*···O14^i^	0.98	2.56	3.465 (2)	154
C18—H18*C*···O17^ii^	0.98	2.63	3.488 (2)	146
C15—H15*A*···*Cg*1^iii^	0.98	2.84	3.692 (2)	139
C12—H12*A*···*Cg*2^iv^	0.98	3.19	4.061 (2)	132
C18—H18*B*···*Cg*1^v^	0.98	3.01	3.976 (2)	149

Symmetry codes: (i) *x*−1/2, −*y*+1/2, −*z*; (ii) *x*−1/2, *y*, −*z*+1/2; (iii) *x*−1/2, −*y*+3/2, −*z*+1; (iv) *x*+1/2, −*y*+1/2, −*z*; (v) −*x*+3/2, −*y*+1, *z*−1/2.

## References

[bb1] Dolomanov, O. V., Bourhis, L. J., Gildea, R. J., Howard, J. A. K. & Puschmann, H. (2009). *J. Appl. Cryst.* **42**, 339–341.

[bb2] Lahtinen, M., Nättinen, K. & Nummelin, S. (2013*a*). *Acta Cryst.* E**69**, o383. 10.1107/S1600536813004133 PMC3588494 23476569

[bb3] Lahtinen, M., Nättinen, K. & Nummelin, S. (2013*b*). *Acta Cryst.* E**69**, o460. 10.1107/S1600536813005333 PMC3588445 23476626

[bb4] Lahtinen, M., Nättinen, K. & Nummelin, S. (2013*c*). *Acta Cryst.* E**69**, o510–o511. 10.1107/S1600536813006053 PMC3629539 23634057

[bb5] Lahtinen, M. & Nummelin, S. (2013). *Acta Cryst.* E**69**, o681. 10.1107/S1600536813008957 PMC3647875 23723841

[bb6] Macrae, C. F., Edgington, P. R., McCabe, P., Pidcock, E., Shields, G. P., Taylor, R., Towler, M. & van de Streek, J. (2006). *J. Appl. Cryst.* **39**, 453–457.

[bb7] Mekelburger, H.-B., Rissanen, K. & Vögtle, F. (1993). *Chem. Ber.* **126**, 1161–1169.

[bb8] Nättinen, K. & Rissanen, K. (2003). *Cryst. Growth Des.* **3**, 339–353.

[bb9] Nonius (1998). *COLLECT* Nonius BV, Delft, The Netherlands.

[bb10] Nummelin, S., Skrifvars, M. & Rissanen, K. (2000). *Top. Curr. Chem.* **210**, 1–67.

[bb11] Otwinowski, Z. & Minor, W. (1997). *Methods in Enzymology*, Vol. 276, *Macromolecular Crystallography*, Part A, edited by C. W. Carter Jr & R. M. Sweet, pp. 307–326. New York: Academic Press.

[bb12] Percec, V., Holerca, M. N., Nummelin, S., Morrison, J. J., Glodde, M., Smidrkal, J., Peterca, M., Uchida, S., Balagurusamy, V. S. K., Sienkowska, M. J. & Heiney, P. A. (2006). *Chem. Eur. J.* **12**, 6216–6241. 10.1002/chem.200600178 16841348

[bb13] Percec, V., Peterca, M., Dulcey, A. E., Imam, M. R., Hudson, S. D., Nummelin, S., Adelman, P. & Heiney, P. A. (2008). *J. Am. Chem. Soc.* **130**, 13079–13094. 10.1021/ja8034703 18771261

[bb14] Percec, V., Smidrkal, J., Peterca, M., Mitchell, C. M., Nummelin, S., Dulcey, A. E., Sienkowska, M. J. & Heiney, P. A. (2007). *Chem. Eur. J.* **13**, 3989–4007. 10.1002/chem.200601582 17304597

[bb15] Percec, V., Wilson, D. A., Leowanawat, P., Wilson, C. J., Hughes, A. D., Kaucher, M. S., Hammer, D. A., Levine, D. H., Kim, A. J., Bates, F. S., Davis, K. P., Lodge, T. P., Klein, M. L., DeVane, R. H., Aqad, E., Rosen, B. R., Argintaru, A. O., Sienkowska, M. J., Rissanen, K., Nummelin, S. & Ropponen, J. (2010). *Science*, **328**, 1009–1014. 10.1126/science.1185547 20489021

[bb16] Roche, C. & Percec, V. (2013). *Isr. J. Chem.* **53**, 30–44.

[bb17] Ropponen, J., Nättinen, K., Lahtinen, M. & Rissanen, K. (2004*a*). *CrystEngComm*, **6**, 559–566.

[bb18] Ropponen, J., Nummelin, S. & Rissanen, K. (2004*b*). *Org. Lett.* **6**, 2495–2497. 10.1021/ol049555f 15255674

[bb19] Ropponen, J., Tuuttila, T., Lahtinen, M., Nummelin, S. & Rissanen, K. (2004*c*). *J. Polym. Sci. Part A Polym. Chem.* **42**, 5574–5586.

[bb20] Sheldrick, G. M. (1996). *SADABS.* University of Göttingen, Germany.

[bb21] Sheldrick, G. M. (2008). *Acta Cryst.* A**64**, 112–122. 10.1107/S0108767307043930 18156677

